# Supplementary material for: Genome-Wide Identification and Analysis of MYB Transcription Factors in Pyropia yezoensis
Source: Plants (Basel). 2023 Oct 19;12(20):3613. doi: 10.3390/plants12203613 (PMC10609806; doi:10.3390/plants12203613)
Supplement: Supplementary file 1 [file plants-12-03613-s001.zip › Supplementary Table S1.pdf]

Supplementary Table S1. Gene primers designed for qRT-PCR.

**Primers**	**Primer sequences (5' to 3')**
PyCGS1-F	CTACGGACACCAAGAAACG
PyCGS1-R	CTCGGTTGGCTGGGTAA
PyUBC-F	TCACAACGAGGATTTACCACC
PyUBC-R	GAGGAGCACCTTGGAAACG
PyMYB2-F	GGCAAGACCCGCAAGAGC
PyMYB2-R	CGAGATGCCACTCTTGACCC
PyMYB3-F	GGGACGTGGACGTGTGG
PyMYB3-R	GAGGGAGGGAGGGCTGA
PyMYB4-F	CGTCTACCGCCTTCCGTG
PyMYB4-R	CTGCTGCTCCTGCCTCATC
PyMYB5-F	GACCTCAAGACCGTTCCCAA
PyMYB5-R	CTCCGAATACCGCTCCTCAC
PyMYB6-F	ATTGAGGATGCGTTTTTAGGAGA
PyMYB6-R	TTGCCGAGTAGAGGGTGAGC
PyMYB7-F	ACCTACCTCCCCTATGATGACG
PyMYB7-R	AACAAACTGCTGGTGCTCTGC
PyMYB8-F	AAGGAGTTTGTGGTCAGCCG
PyMYB8-R	CCCCGTCAGAATGGAATCG
PyMYB9-F	ACGGCGCAAGAGAAGGAGT
PyMYB9-R	GGAAGGACGGTCTGGGAAT
PyMYB12-F	CGAGGAAAGTACACCCTAGCC
PyMYB12-R	CCAGATTAGAGACAGATCCGAAA
PyMYB16-F	ACAGCCAGACATACCAACCCA
PyMYB16-R	CAACCGAGACATCAGCACCA
