# Supplementary material for: Genome-Wide Identification and Analysis of MYB Transcription Factors in Pyropia yezoensis
Source: Plants (Basel). 2023 Oct 19;12(20):3613. doi: 10.3390/plants12203613 (PMC10609806; doi:10.3390/plants12203613)
Supplement: Supplementary file 1 [file plants-12-03613-s001.zip › Supplementary Table S2.pdf]

Supplementary Table S2.Number of MYB genes in examined species.

Taxonomic group	Species name	MYB genes				Total	Genome size (Mb)	Genome accesion in NCBI database
		1R	R2R3	3R	4R			
Rhodophyta (Red algae)	Pyropia yezoensis	13	3	0	0	16	107.6	GCA_009829735.1
	Pyropia haitanensis	9	4	0	0	13	53.3	GCA_008729055.1
	Porphyra umbilicalis	8	6	0	0	14	87.9	GCA_002049455.2
	Chondrus crispus	15	4	1	1	21	105.0	GCF_000350225.1
	Gracilariopsis chorda	20	6	1	0	27	92.2	GCA_003194525.1
	Porphyridium purpureum	32	17	1	0	50	19.7	GCA_008690995.1
	Cyanidioschyzon merolae	17	5	0	1	23	16.4	GCF_000091205.1
	Galdieria sulphuraria	16	7	2	0	25	14.3	GCF_000341285.1
Glaucophyta	Cyanophora paradoxa	12	3	0	0	15	99.9	GCA_004431415.1
Chlorophyta (Green algae)	Chlamydomonas reinhardtii	14	10	1	0	25	111.1	GCF_000002595.1_v3.0
	Micromonas pusilla	19	7	1	0	27	22.0	GCF_000151265.2
Charophyta (Green algae)	Chara braunii	9	9	2	1	21	1,751.2	GCA_003427395.1
Embryophyta	Arabidopsis thaliana	64	126	5	1	196	115.4	Ref. (Chen, Yang et al. 2006)
