# Supplementary material for: Genome-Wide Identification and Analysis of MYB Transcription Factors in Pyropia yezoensis
Source: Plants (Basel). 2023 Oct 19;12(20):3613. doi: 10.3390/plants12203613 (PMC10609806; doi:10.3390/plants12203613)
Supplement: Supplementary file 1 [file plants-12-03613-s001.zip › Supplementary Table S5.pdf]

Supplementary Table S5. Ka, Ks and Ka/Ks values for duplicated ortholog pairs in *Pyropia yezoensis* and *Pyropia haitanensis*/*Porphyra umbilicalis*.

No.	Seq 1	Seq 2	Ka	Ks	Ka/Ks
1.	*PyMYB1*	*Ph05488*	0.1930	0.8942	0.2158
2.	*PyMYB2*	*Ph05827*	0.0553	0.3528	0.1567
3.	*PyMYB3*	*Ph04980*	0.0780	0.4278	0.1823
4.	*PyMYB4*	*Ph09887*	0.1696	0.5794	0.2927
5.	*PyMYB5*	*Ph02171*	0.0328	0.4024	0.0815
6.	*PyMYB6*	*Ph02359*	0.1479	0.5822	0.2540
7.	*PyMYB7*	*Ph08492*	0.2697	0.5027	0.5365
8.	*PyMYB8*	*Ph06857*	0.0520	0.4748	0.1095
9.	*PyMYB9*	*Ph07942*	0.1887	0.7457	0.2531
10.	*PyMYB10*	*Ph07589*	0.2640	0.8541	0.3091
11.	*PyMYB12*	*Ph05957*	0.5541	0.9707	0.5708
12.	*PyMYB14*	*Ph05025*	0.1970	0.5709	0.3451
13.	*PyMYB16*	*Ph09161*	0.1280	0.5195	0.2464
14.	*PyMYB2*	*PuOSX69724*	0.1411	0.7978	0.1769
15.	*PyMYB3*	*PuOSX72389*	0.0875	0.4807	0.1820
16.	*PyMYB5*	*PuOSX76044*	0.1578	1.0509	0.1502
17.	*PyMYB6*	*PuOSX79855*	0.2451	1.3556	0.1808
18.	*PyMYB8*	*PuOSX75147*	0.1132	1.2917	0.0876
