# Supplementary figures and images for: Genome-Wide Identification and Analysis of MYB Transcription Factors in Pyropia yezoensis
Source: Plants (Basel). 2023 Oct 19;12(20):3613. doi: 10.3390/plants12203613 (PMC10609806; doi:10.3390/plants12203613)

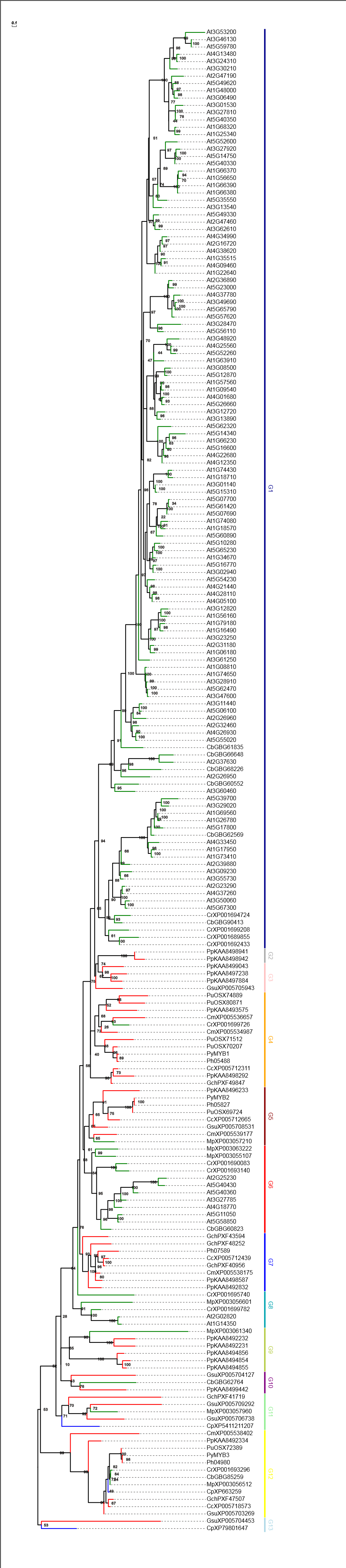

Supplement: Supplementary file 1 [file plants-12-03613-s001.zip › Figure S1.png]

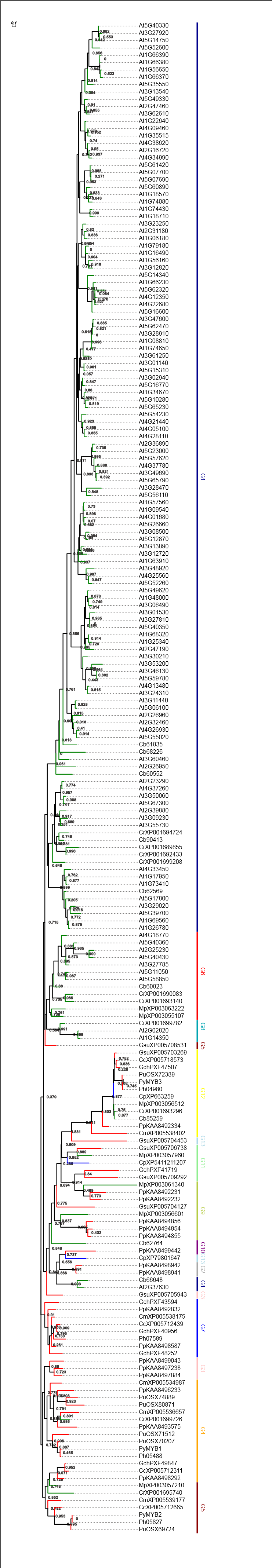

Supplement: Supplementary file 1 [file plants-12-03613-s001.zip › Figure S2.png]

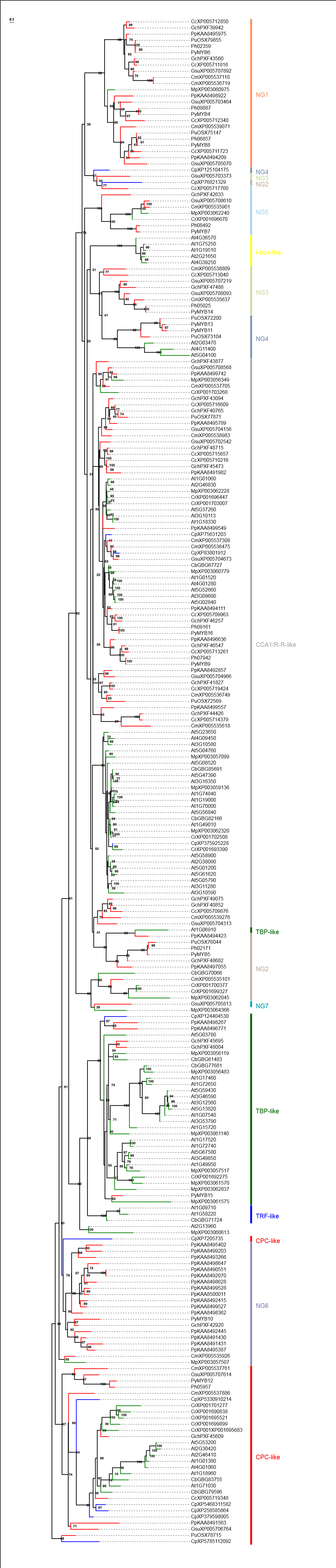

Supplement: Supplementary file 1 [file plants-12-03613-s001.zip › Figure S3.png]

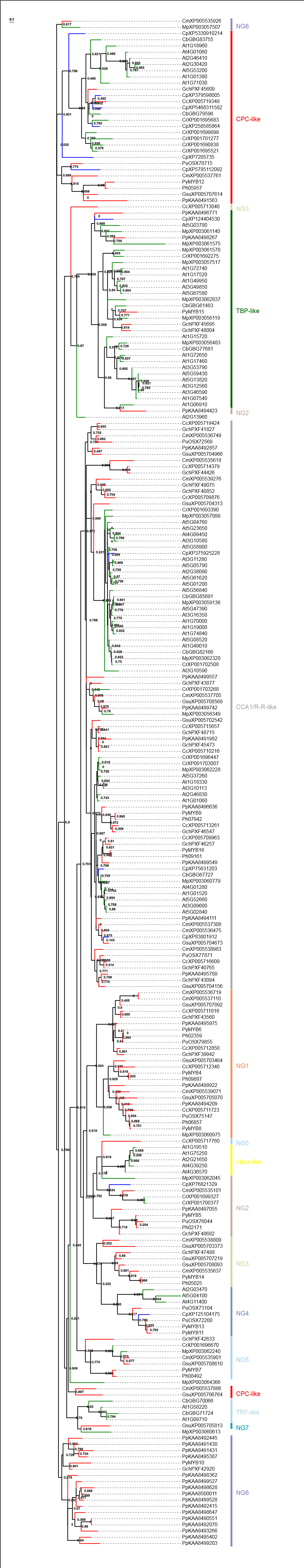

Supplement: Supplementary file 1 [file plants-12-03613-s001.zip › Figure S4.png]
